# Chronic Primary Pelvic Pain Syndromes in Women: A Comprehensive Review

**DOI:** 10.7759/cureus.74918

**Published:** 2024-12-01

**Authors:** Luisa Pinto, Mariana Soutinho, Manuel Coutinho Fernandes, Maria Inês Táboas, Joana Leal, Sónia Tomé, Jorge Moreira, Ana Zão

**Affiliations:** 1 Physical Medicine and Rehabilitation, Unidade Local de Saúde de Entre Douro e Vouga, Santa Maria da Feira, PRT; 2 Physical Medicine and Rehabilitation, Unidade Local de Saúde de Trás-os-Montes e Alto Douro, Vila Real, PRT; 3 Physical Medicine and Rehabilitation and Chronic Pain, Unidade Local de Saúde de Santo António, University of Porto, Porto, PRT

**Keywords:** anorectal diseases, chronic pelvic pain syndrome, rehab, sexual problems, treatment choices

## Abstract

Chronic pelvic pain (CPP) in women is a multifactorial and complex condition. It often remains undiagnosed or inadequately treated. Despite its high prevalence, CPP continues to be a taboo subject, leading to delays in seeking medical care. Chronic primary pelvic pain syndromes (CPPPS) are pain conditions without an obvious underlying diagnosis, including painful bladder syndrome, vulvodynia, genito-pelvic pain/penetration disorder, levator ani syndrome, proctalgia fugax, myofascial syndrome, pudendal neuralgia, and coccyx pain syndrome.

A comprehensive review of the literature was conducted to understand the most common forms of CPPPS in women, focusing on diagnostic criteria, pathophysiology, and treatment options. Due to the complexity of CPPPS and varied treatment responses, management requires a multidisciplinary approach. Although various treatment modalities exist, no single strategy is universally effective, emphasizing the need for individualized care. Future research should prioritize refining diagnostic criteria and investigating new therapeutic strategies.

## Introduction and background

Chronic pelvic pain (CPP) is characterized by a multifactorial nature, with various types of pain potentially coexisting [1]. Therefore, around 60% of women with this condition never receive a precise diagnosis, and about 20% do not undergo any investigation to determine the cause of their pain [1]. Despite its prevalence, CPP remains a taboo subject, resulting in delayed help-seeking behaviors [2].

CPP is defined by the European Association of Urology (EAU) as persistent pain for at least six months perceived in pelvic structures among both men and women, which often manifests with negative cognitive, behavioral, sexual, and emotional consequences, alongside symptoms suggestive of the lower urinary tract, sexual, bowel, pelvic floor, or gynecological dysfunction [3].

The EAU assorts CPP with no obvious diagnosis, known as chronic primary pelvic pain syndromes (CPPPS), and non-pain syndromes, which have an identifiable underlying pathology such as infection, neuropathy, or inflammation [3].

**Figure 1:**
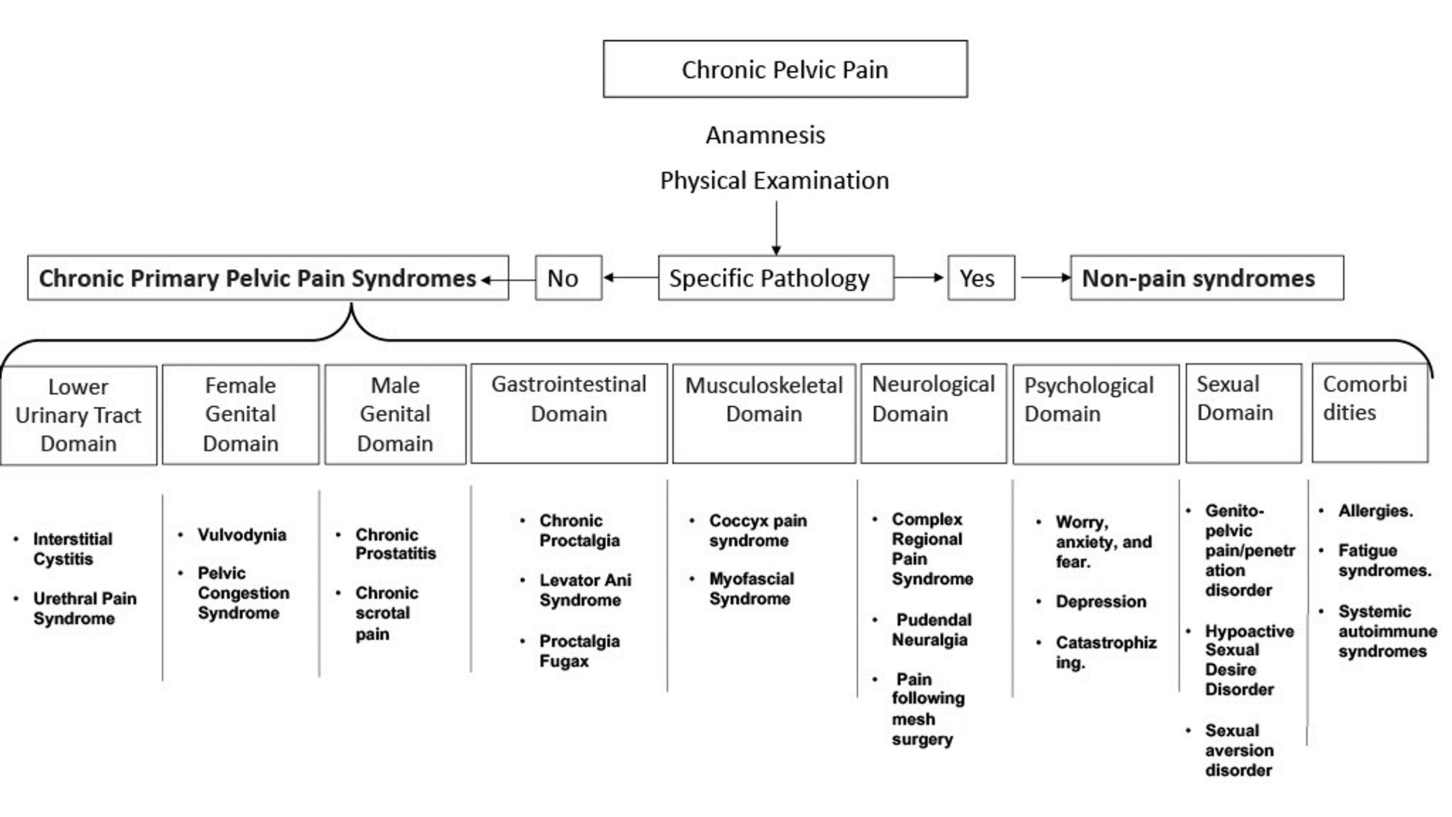
Diagram of chronic pelvic pain, detailing the nine major groups of chronic primary pelvic pain syndromes

In 2017, a working group of the International Continence Society (ICS) outlined nine major groups crucial for diagnosing CPPPS: lower urinary tract domain, female genital domain, male genital domain, gastrointestinal domain, musculoskeletal domain, neurological domain, psychological domain, sexual domain, and comorbidities (Figure 1) [4]. In this review, we will focus on discussing the most prevalent categories of CPPPS in women. The aim is to critically assess the current understanding of the diagnosis and treatment of the most common forms of CPPPS in women. This article is intended to assist physicians in managing complex consultations related to CPPPS.

## Review

Interstitial cystitis/painful bladder syndrome (IC/PBS): lower urinary tract domain

PBS, also known as IC, is defined by persistent pain perceived in the bladder, along with urinary frequency and/or urgency, with unknown etiology [5]. The prevalence ranges from 0.83% to 2.71%, with higher occurrence in women (89.8%) and in the Caucasian race (94.1%) and symptom onset typically around age 30 [6]. Some cases present with changes in the urothelium such as glomerulations and Hunner's ulcers. However, glomerulations are not undoubtedly pathological [7].

The symptoms of IC/PBS result from bladder sensory dysregulation, leading to increased pain perception, as well as heightened sensations of urgency and/or urinary frequency. It is a diagnosis of exclusion. Therefore, the investigation of other potential causes that could justify the symptoms, such as acute or recurrent infections, radiation- or medication-induced injuries, tumors, or nephrolithiasis, is needed. Although diagnostic criteria have evolved over the years, there is still no consensus on its definition [6]. The most widely used definitions are those of the European Society for the Study of IC/PBS (CPP, pressure, or discomfort perceived as related to the bladder, accompanied by one or more urinary symptoms, considered a diagnosis of exclusion lasting at least six months, and requiring cystoscopy for diagnosis [8]) and the American Urological Association (an unpleasant sensation, pain, pressure, or discomfort, perceived as related to the bladder, along with symptoms of the lower urinary tract, requiring the exclusion of other etiologies, and lasting for six weeks) [9].

The pathophysiology of this condition involves alterations in the protective layer of glycosaminoglycans, which regulates the passage of cations and protects the urothelium from harmful substances and bacteria [6]. It is also thought that there is an inadequate activation of mast cells, which are found in greater numbers in the urothelium of these patients. Estrogen receptors also play a role, as increased estrogen levels can lead to histamine degranulation and the subsequent release of substance P, which acts as a neuropeptide that promotes inflammation, pain signaling, and increased vascular permeability [6]. The association of urinary tract infection with this condition is suggested by a high number of positive urine cultures, altered urinary bacterial flora, or whole transcriptome analysis. An increased frequency of Epstein-Barr virus infection has also been reported in patients with IC/PBS with Hunner's ulcers [7].

Treatment should begin with behavioral and dietary modifications, such as avoiding foods that exacerbate symptoms, such as citrus fruits, caffeine, alcoholic beverages, and spicy foods, as well as behavioral factors that aggravate symptoms like stress, constrictive clothing, and sexual intercourse [10]. As a second-line treatment, pelvic floor rehabilitation aims to release myofascial trigger points (MTrPs), release connective tissue, and train muscular coordination, resulting in symptomatic improvement in over 50% of patients [6].

Pharmacological treatment should be initiated when lifestyle changes are insufficient and can be implemented concomitantly with rehabilitation as an adjuvant therapy. Amitriptyline (tricyclic antidepressant) has shown significant improvement in symptoms, ranging from 50% to 77% [6]. Standard dosing starts at 10 mg daily with gradual increases to a maximum of 100 mg as tolerated [11]. Hydroxyzine is an FDA-approved antihistamine and mast cell stabilizer for the treatment of IC/PBS. It has shown effectiveness in providing significant symptom relief for some IC/PBS patients, especially those with allergies. It prevents mast cell degranulation, reducing the release of inflammatory agents. Adverse effects are typically minor, including mild sedation and weakness [12]. Sodium pentosan polysulfate reduces urothelial permeability by reinforcing the glycosaminoglycan layer [7]. It improves frequency and pain, especially in patients with ulcerative IC/PBS, and is well-tolerated [6]. However, studies on its efficacy are conflicting [13].

Dimethyl sulfoxide is the only FDA-approved intravesical treatment. It has anti-inflammatory action, dissolves collagen, and relaxes the detrusor [11]. This therapeutic option should be considered for patients who have not responded adequately to lifestyle changes, rehabilitation, or oral medications and is the only intravesical treatment classified as a second-line therapy [6]. Some patients may not tolerate the pain or odor associated with it [6]. Hydrodistension with or without instillation has been shown to be effective and safe. Some studies reported an efficacy rate of approximately 50% [14], with effects lasting from a few months to a long-term efficacy of over one year [7]. Longer duration has been found in patients with ulcerative IC/PBS and in patients without other comorbidities [6]. Fulguration is recommended for IC/PBS with Hunner's ulcers, as it improves symptoms in over 90% of patients [6,14], with efficacy lasting for a few months to two years post-treatment. However, nearly half of the patients will require retreatment within 2-5 years [7].

Botulinum toxin type A (BTA) is a neurotoxic protein that can be injected into the detrusor muscle, resulting in symptomatic improvement by flaccid paralysis of this muscle. A recent study showed that at eight weeks, there was a significantly greater reduction of pain in the BTA group compared to the placebo group, and the overall success rate with BTA bladder injections was reported to be over 63% in this condition [15].

Percutaneous tibial nerve stimulation and transcutaneous electrical nerve stimulation (TENS) offer potential benefits, being minimally invasive and more patient-friendly, and therefore should be the first step in neuromodulation [16]. Sacral neuromodulation is effective in some refractory cases for controlling intractable bladder overactivity and CPP in some IC/PBS patients. Several studies have found significant improvement in pain, frequency, voiding volume, and quality of life and decreased opioid use [6,11]. Given the lack of randomized studies, this should be limited to patients with significant and refractory symptoms.

Cyclosporine A (calcineurin inhibitor) is used in rare situations, in intractable cases. It improves quality of life, reduces pain, and increases bladder capacity. The benefits of low-dose cyclosporine A should be weighed against its side effects (hypertension and impaired renal function) [17]. More radical surgical treatment, such as supra-trigonal bladder resection combined with augmentation ileocystoplasty, has been proposed as an effective surgical treatment for many patients, avoiding the necessity for a urostomy [18].

Vulvodynia: female genital domain

The terminology of the 2015 International Society for the Study of Vulvovaginal Disease (ISSVD), the International Society for the Study of Women's Sexual Health (ISSWSH), and the International Pelvic Pain Society (IPPS) consensus defines vulvodynia as vulvar pain lasting for more than three months without an identifiable cause, often associated with other conditions such as musculoskeletal and neurological factors, psychosocial factors, as well as painful comorbidities (including fibromyalgia, irritable bowel syndrome, and migraines, among others) [19].

Vulvodynia is a condition that occurs in 8-10% of women of all ages [20,21]. However, only 60% of affected women seek treatment, and only about 50% of those receive a formal diagnosis of vulvodynia [22].

It's believed that the pathophysiology of vulvodynia involves various factors: injury or irritation of the nerves in the vulvar region leading to increased sensitivity and pain perception [23,24]; previous vaginal infections, most importantly recurrent *Candida* vulvovaginitis, which may contribute to the ongoing inflammation and discomfort in the vulvar area [20]; allergies or sensitive skin which can exacerbate symptoms, as exposure to certain substances may trigger irritation and pain [24]; and hormonal changes, such as low estrogen levels or increased innervation, which can alter the vaginal environment and affect nerve function, potentially leading to vulvar pain [21]. Muscle dysfunction is frequently associated with vulvodynia and is characterized by increased pelvic floor muscle tone, along with changes in muscle contractility and control, as confirmed by 4D ultrasound and dynamometry [25]. Additionally, vulvodynia often coexists with fibromyalgia, further suggesting a role for muscular dysfunction in this condition [25]. Also, vaginal atrophy can result in increased susceptibility to irritation and pain in the vulvar region [26]. A history of physical abuse was associated with a fourfold increased risk of vulvodynia, while a history of sexual abuse was linked to a sixfold increased risk [22]. These factors, either independently or in combination, contribute to the complex pathophysiology of vulvodynia [20].

Generalized vulvodynia affects the entire vulva, but the pain can be specifically localized to the clitoris (clitorodynia) or the vestibule of the vagina (vestibulodynia). Provoked vulvodynia is triggered by contact such as a cotton swab, tampon, or penetration, whereas unprovoked vulvodynia is characterized by pain without any apparent trigger. Additionally, primary vulvodynia is identified by pain since the first attempt at tampon insertion or sexual penetration, while secondary vulvodynia arises after more than three months of pain-free intercourse. Various temporal patterns of the pain, such as intermittent, persistent, constant, immediate, or delayed, are also described [21].

During inspection, mild erythema is characteristic but not pathological [27]. The clitoris should be approximately the size of the head of a cotton swab. A reduction in the size of the clitoral head can indicate hormonal insufficiencies due to oral contraceptive use, hormonal suppressive therapies, or menopause [27]. Hypertonic muscles and tenderness can be detected through firm digital pressure applied to both the levator ani muscle group and the obturator internus [28].

Various techniques have been described for the assessment of vulvar sensitivity. The cotton swab test is a common method for assessing sensitivity. This involves applying light pressure to various regions of the vulvar vestibule with a cotton swab, testing in a clockwise direction, along with bilateral pressure on the hymenal remnants. This test usually elicits tenderness at one or more points in most women with vulvodynia but rarely causes tenderness in women without vulvar pain [20].

Treatment for vulvodynia typically involves a multidisciplinary approach aimed at managing symptoms and improving quality of life. Hygiene guidelines and local moisturizers help maintain vulvar health and reduce irritation. Frequent recommendations include using non-irritating cotton underwear, avoiding vulvar irritating agents, and using non-irritating soaps. Good drying and cleaning of the vulva after micturition is necessary, and good lubrication with non-irritating agents is recommended for sexual intercourse [24].

Topical treatments are appealing because they can be applied to the targeted area and have little systemic absorption. In vulvodynia, there is an increase in unmyelinated C-fibers, which transmit dull, delayed, diffuse, achy, and burning pain, which promotes nerve irritation. Lidocaine blocks the conduction of C-fibers and can also block calcitonin gene-related peptides, calming irritable nociceptors when used continuously [28]. Lidocaine ointment 5% is the most commonly recommended local medication. However, recently, a multicentric study showed that the manual perineal rehabilitation using lidocaine 2% gel had a positive impact on well-being, perceived vulvar pain, perceived sexual function, and dyspareunia. Lidocaine 2% gel was preferred due to previous reports of patients experiencing a severe sensation of vulvar burning after the application of the lidocaine ointment 5% [29]. It is recommended to use topical lidocaine 30 minutes before sexual intercourse or when the patient has complaints. Daily perineal massage can help desensitize the vulva, particularly before sexual activity [24].

Topical estrogen therapy may be recommended for peri- or post-menopausal women to address hormonal imbalances and improve vaginal atrophy [30]. Psychological support and sexual therapy can be beneficial in addressing the emotional impact of vulvodynia and improving sexual function and intimacy [20,21]. Cognitive behavioral therapy is associated with a 30% decrease in reported vulvar pain with intercourse [22,24]. Also, vaginal dilators can desensitize the vulva to touch and pressure and stretch hypertonic pelvic floor muscles and the vagina [31]. However, there are few studies on vaginal dilators, with most of them focusing on post-radiotherapy cases.

Pelvic floor exercises help strengthen pelvic muscles and may aid in pain management by identifying and addressing contributing factors [28]; TENS inhibits pain through gate control theory and by stimulating the release of endogenous opioids. A double-blind placebo randomized controlled trial (RCT) compared intravaginal TENS with a sham TENS, finding that TENS significantly reduced pain and dyspareunia [32].

Pharmacotherapy is often used for symptom relief. Recommended medications include tricyclic antidepressants, such as amitriptyline as a first-line treatment and pregabalin/gabapentin (gabapentin from 300 to 1500 mg daily or pregabalin from 50 to 150 mg daily) as second-line options, as recommended by the Vulvodynia Network Group [33].

It is known that BTA inhibits the release of glutamate and substance P from nociceptive neurons, potentially reducing peripheral and central sensitization in vulvodynia. The efficacy of BTA for vulvodynia has been evaluated and showed contrasting results [34]. A recent double-blind placebo-controlled RCT performed 50 units of onabotulinumtoxin A twice or placebo in bulbospongiosus. At the 12-month follow-up, no significant difference was observed in the reduction of dyspareunia or pain during tampon use. However, women who received BTA attempted intercourse more frequently and experienced improvements in their sexual function compared to those who received a placebo [35]. Another double-blind placebo-controlled RCT showed that provoked vestibulodynia symptoms after one subcutaneous injection of BTA (50 or 100 units of onabotulinumtoxin A) did not significantly differ compared to placebo (saline), yet all three study arms experienced a reduction in pain three months after a single injection [36]. On the other hand, Pelletier et al. injected 100 units of onabotulinumtoxin A, using electromyography guidance, in the superficial pelvic floor muscles of 20 women with vulvodynia, resulting in a statistically significant pain reduction at six months after injection [37]. Thus, BTA is not recommended as a first-line treatment but can be considered a second-line option, pending further trials.

These treatment modalities are often used in combination to provide comprehensive care tailored to the individual needs of each patient [20,25]. Vestibulectomy is recommended as a management option, once other less invasive treatment options have been attempted [20]. The success of vestibulectomy varies between 60% and 90% in comparison with the success of non-surgical methods, between 40% and 80% [24].

Genito-pelvic pain/penetration disorder (GPPPD): sexual domain

GPPPD is a diagnosis introduced in the Fifth Edition of the Diagnostic and Statistical Manual of Mental Disorders (DSM-5). This diagnosis combines the previously separate conditions of dyspareunia and vaginismus. GPPPD is characterized by at least one of the following persistent or recurrent symptoms: difficulties with vaginal penetration during intercourse, genito-pelvic pain during intercourse or attempts at penetration, fear or anxiety related to genito-pelvic pain or vaginal penetration, or marked tension or contraction of the pelvic floor muscles during attempted penetration [38]. The DSM-5 criteria specify that symptoms must be present for approximately six months, causing clinically significant distress to the patient and not being better explained by a non-sexual mental disorder.

Although vaginismus and dyspareunia are included under the umbrella of GPPPD in the DSM-5, it is still common to refer to each condition separately. Vaginismus typically refers to involuntary muscle spasms that prevent vaginal penetration, while dyspareunia involves pain during intercourse [39]. Reported prevalence rates in the general population vary between 3% and 25% for dyspareunia and 0.4% and 6.6% for vaginismus [40]. However, because of cultural differences, the prevalence is very different in undeveloped and developed countries. In underdeveloped countries, most women do not report their pain or seek treatment because of shame or other cultural factors such as gender superiority [41].

Additionally, a recent systematic review found that the prevalence of dyspareunia is 42% at two months postpartum, 43% at 2-6 months postpartum, and 22% at 6-12 months postpartum. Given these high prevalence rates and their significant impact on a woman's life, the study highlighted the need for special attention to dyspareunia during the postpartum period [42].

Regarding vaginismus, Lamont's grading system (Table 1) categorizes the severity into four grades [43].

**Table 1:** Lamont's grading system for vaginismus

Grades	Description
Grade 1	It is the mildest form, where penetration may be uncomfortable or difficult but is possible with patience and reassurance
Grade 2	It is a moderate form, where penetration requires significant effort and persistence, causing discomfort or pain
Grade 3	It is a severe form, characterized by a complete inability to achieve penetration due to involuntary pelvic floor muscle contractions, making any attempt extremely painful or impossible
Grade 4	It is the most extreme form, where even attempts at gynecological examinations or tampon insertion cause resistance and pain, often leading to the avoidance of any vaginal penetration

Several approaches can be beneficial for treating sexual pain. The treatment is very similar to that for vulvodynia. The use of anesthetic creams, sitz baths, and lubricants can provide some relief [24]. Pelvic floor physical therapy aims to reduce hypertonicity or tension in the pelvic floor muscles, which contribute to sexual pain and make intercourse painful or difficult. This therapy increases awareness of the pelvic muscles, improves relaxation techniques, normalizes muscle tone, and provides stretching stimuli at the introitus to gradually reduce anxiety surrounding penetration. The approach is often multimodal, incorporating techniques such as electromyographic biofeedback, electrical stimulation, manual tissue manipulation, stretching/strengthening exercises, and the use of dilators [44]. For those suffering from vaginismus, self-treatment with dilators combined with lidocaine gel can be beneficial [45]. In cases of vaginal atrophy, the application of topical estrogen is recommended [30] and also lubricants and dilation because of dryness and tearing [45].

Psychological intervention is an important therapy arm, especially in vaginismus, and it aims to explore a woman's thoughts, emotions, behaviors, and relationship dynamics associated with her experience of sexual pain. Research supports the effectiveness of individual cognitive behavioral therapy, showing improvements in pain and sexual function maintained at one year when compared to individual supportive therapy [46].

The O-Shot therapy involves using platelet-rich plasma (PRP) as a novel non-surgical outpatient treatment to improve urinary incontinence and sexual dysfunction. This method uses a woman's own growth factors, with PRP injected into specific areas of the vagina using local anesthetic cream. PRP activates tissue regeneration, leading to significant enhancements in sexual response, such as improved arousal, stronger orgasms, decreased dyspareunia, and increased natural lubrication [47]. In a study by Runels et al. [48], 11 women with dyspareunia received PRP injections in the clitoris and vagina. The results indicated that intravaginal and intraclitoral PRP injections could effectively treat various aspects of female sexual dysfunction, particularly in desire, arousal, lubrication, and orgasm. However, existing studies are not robust, indicating a need for more comprehensive research on this treatment [48].

As with vestibulodynia, injections of BTA have been tested in the treatment of vaginismus, but the evidence for their benefit is modest, and they are not recommended as a first-line treatment but can be considered a second-line option, pending further trials [49].

Levator ani syndrome: gastrointestinal domain

Based on the Rome IV diagnostic criteria, the diagnosis of levator ani syndrome requires that the following conditions be met over the past three months, with symptom onset at least six months prior to the diagnosis: recurrent rectal pain or discomfort, episodes lasting for 30 minutes or more, pain upon the traction of the puborectalis muscle, and the exclusion of other causes of rectal pain [50]. Some patients describe the sensation as feeling like they are sitting on a ball or having a ball inside their rectum [51].

The pathophysiology of levator ani syndrome is most commonly attributed to transient spasms of the pelvic floor muscles, an increase in the resting pressure of the anal canal, and defecatory dyssynergia [50]. Clinically, patients with levator ani syndrome often present with complaints of constipation, pain following defecation, and pain while sitting. On physical examination, an increase in the tone of the levator ani muscle may be observed [52].

In the absence of a well-defined pathophysiological mechanism for these alterations, various therapeutic approaches have been explored. An uncontrolled study found that sitz baths with hot water at 40°C significantly reduce anal pressure [53]. Furthermore, the rehabilitation programs differ from traditional pelvic floor physical therapy for prolapse or incontinence, which primarily targets muscle strengthening of the pelvic floor. In contrast, programs for levator ani syndrome emphasize techniques such as myofascial release, muscle stretching, and posture improvement. Also, biofeedback to improve rectoanal coordination should be the first-line treatment for dyssynergic defecation [51].

If symptoms continue after conservative treatment, a high dose (totaling 200 units of onabotulinumtoxin A) of BTA can be injected into the levator muscle [51]. However, it should be considered an adjunct to conservative therapy [51], given that the supporting evidence is weak, as demonstrated in an RCT of intra-anal BTA injection, which compared it to placebo with saline in 12 patients, showing no difference in pain outcomes between the groups [54].

Proctalgia fugax: gastrointestinal domain

Proctalgia fugax is a benign but often distressing condition characterized by sudden, intense, and fleeting episodes of anal or lower rectal pain [55]. The pain can be severe enough to cause significant discomfort and anxiety. It is a relatively common condition, although the exact prevalence is difficult to determine due to underreporting [56]. It affects both men and women, with some studies suggesting a slightly higher prevalence in women [57]. It can occur at any age, but it is rare before puberty [52].

The diagnosis of proctalgia fugax is primarily clinical, based on the characteristic presentation of sudden, short-lived rectal pain. It is defined by sudden, severe pain in the rectal area, unrelated to defecation, lasting from a few seconds to several minutes (rarely up to 30 minutes), which disappears completely, with other causes of rectal pain excluded [58]. Hereditary proctalgia fugax is associated with constipation and hypertrophy of the internal anal sphincter [52].

Episodes are sporadic, with pain occurring unpredictably at irregular intervals, ranging from one to 180 times per year [59], and typically occurring fewer than five times per year in 51% of patients [50]. Prolonged sitting, sexual intercourse, and stress can serve as predisposing factors, though most patients do not report specific triggering events [59]. While it was previously believed that pain episodes occur predominantly at night, a recent prospective study of 54 patients with proctalgia fugax did not support this, suggesting instead that patients may simply recall nocturnal attacks more vividly; this study also found that the average pain duration was 15 minutes [59].

Due to the transient nature of the symptoms, treatment is usually unnecessary [51]. However, for patients who suffer from more frequent and prolonged symptoms, several treatment options may be considered to alleviate discomfort and improve quality of life. Since the primary etiology is suggested to be intermittent anal spasm, treatments that induce the relaxation of the internal anal sphincter have been utilized in the therapeutic arsenal. Unfortunately, there are only a few case series and a single RCT that guide the management of these patients [60]. Warm sitz baths, warm tap water enemas, and digital anal dilatation are often suggested as simple interventions to achieve rapid sphincter relaxation [60]. Oral diazepam, commonly prescribed as a muscle relaxant, is used as a short-term treatment. Glycerin suppositories are another option for managing proctalgia fugax symptoms by relaxing the muscles in the anal canal, thus helping to alleviate discomfort, but the effectiveness of these treatments is supported only by case reports or case series, with the exception of a single RCT of salbutamol [55]. Inhalation of salbutamol, a β2 adrenergic agonist, has shown promise in relieving symptoms by relaxing the smooth muscles of the intestines and potentially decreasing the duration and severity of painful episodes, with an RCT demonstrating its greater effectiveness compared to placebo in shortening the episodes of proctalgia lasting 20 minutes or longer [61]. In cases where first-line treatments fail to provide adequate relief, BTA injections may be considered as a second-line therapy for proctalgia fugax. A recent case report showed that after two administrations of BTA, 80 units and 100 units each (the type of BTA was not specified), the patient remained asymptomatic at the eight-month follow-up [62]. However, there is limited research about BTA in patients suffering from this condition.

Coccyx pain syndrome (CPS): musculoskeletal domain

Coccygodynia is characterized by pain felt in the region of the coccyx, which is exacerbated by pressure or the manipulation of the coccyx. Symptoms typically include sacrococcygeal or anal pain, which worsens when sitting, when leaning backward due to compression, or during defecation [63]. Coccygodynia may occur due to traumatic, nontraumatic, or idiopathic causes known as CPS. The most common cause of coccygodynia is direct trauma [64].

Managing this condition can be challenging due to the complex nature of coccygeal pain [65]. Most cases resolve within a few weeks to months, but for some patients, the pain can become chronic and negatively impact their quality of life [65].

Both obesity and female sex are known predisposing factors for coccygodynia. A body mass index of over 27.4 in women and 29.4 in men is a risk factor. The higher incidence in obese individuals is attributed to restricted sagittal pelvic rotation while sitting, causing protrusion and excessive pressure on the coccyx [63]. Women are five times more prone to coccygodynia due to ligamentous laxity, susceptible coccygeal morphology, and childbirth [63]. Increased intrapelvic pressure during sitting can also lead to posterior subluxation of the coccyx tip [63]. Intercoccygeal articulations are known to contain various intervertebral disc variants. Degenerative changes in these discs or their variants have been implicated as a cause of pain in 41% of CPS cases [66]. CPS can also arise from the instability of the coccyx with or without pelvic floor spasm [51].

Diagnosis of coccygodynia is clinical, which may reveal tenderness upon the palpation of the coccyx and hyper- or hypomobility of the coccyx (the normal range of motion should be approximately 13 degrees) [65]. Imaging studies such as dynamic X-rays and magnetic resonance imaging may be used to evaluate for coccyx luxation or other structural abnormalities contributing to the pain. The normal morphology of the coccyx is highly variable. Postacchini and Massobrio initially classified these morphological variants into four types, to which Nathan et al. later added two more types [67,68]. Types III to VI were found to have a significantly higher incidence in patients with coccygodynia [63]. Lateral views are always indicated as coccyx curvature can be classified into different types [67]. Table 2 lists the different types of coccyx curvature.

**Table 2:** Different types of coccyx curvature

Type	Description
Type I	The coccyx is slightly curved forward
Type II	The coccyx is pointed straight forward
Type III	The coccyx has a sharp forward angulation
Type IV	The coccyx shows subluxation at the sacrococcygeal or intercoccygeal joint
Type V	The coccyx is retroverted with a posteriorly angulated apex
Type VI	The coccyx is scoliotic or laterally subluxated

Conservative treatment is generally effective in about 90% of the cases [65]. The treatment can involve several strategies. Analgesia with nonsteroidal anti-inflammatory drugs (NSAIDs) is often recommended to reduce inflammation and relieve pain associated with coccygodynia. Opioid medications may be prescribed, particularly in cases of severe pain. In patients with pelvic hypertonia or muscle spasms, muscle relaxants and benzodiazepines are also prescribed [69].

For patients experiencing constipation, laxatives may be prescribed to soften the stool and ease bowel movements, thereby reducing pressure on the coccyx during defecation [64]. Donut-shaped cushions are commonly used to relieve coccyx pressure while sitting, as they help distribute weight more evenly and alleviate discomfort from prolonged sitting [70].

Pelvic floor rehabilitation can be beneficial for coccygodynia associated with pelvic floor muscle spasms. Thiele described the soft tissue mobilization technique for the levator ani, coccygeus, and piriformis muscles, known as Thiele's massage. This technique is performed intrarectally using a single finger. The stroke starts just lateral to the coccyx, sweeps laterally, and then moves anteriorly and finally medially, with 10-15 repetitions, performed bilaterally. Soft tissue mobilization to the anal rim targeting the external anal sphincter is also helpful to improve soft tissue mobility in the presence of scarring [63,64]. Intrarectal manipulation can identify and potentially correct a dislocated sacrococcygeal joint [71]. A study showed that patients with an immobile coccyx had the poorest outcomes with these manual techniques, while those with a normally mobile coccyx experienced the best results [71].

TENS can be advantageous, using either an external technique with two cutaneous probes or an internal technique with one cutaneous probe and one intrapelvic probe [65].

In cases where conservative measures fail to provide sufficient relief, second-line treatments may be considered [63]. This may include corticosteroid-anesthetic injections, which can temporarily alleviate pain by reducing inflammation and blocking pain signals [70]. Another option is the blockade of the ganglion impar, a cluster of nerve cells near the coccyx, which can be targeted with medications or nerve blocks to help alleviate pain in refractory cases [72].

The efficacy of coccygectomy for coccygodynia varies, with success rates ranging from 60% to 91%, with better outcomes in patients with sacrococcygeal instability and hypermobility. Also, it seems that patients with moderate to severe degenerative changes in the sacrococcygeal joint had better postoperative outcomes [70].

Myofascial syndrome in pelvic pain: musculoskeletal domain

Myofascial pain syndrome (MPS) in the pelvic floor musculature is one of the most common causes of CPP in women [73], and it is characterized by the presence of MTrPs, which are hard, palpable, localized nodules in taut bands of muscles that are painful upon compression and may produce characteristic referred pain, referred tenderness, motor dysfunction, and autonomic phenomena [73,74].

It can be either a primary condition or a secondary manifestation resulting from another underlying disease. When dealing with secondary myofascial pain, it is essential to treat the primary pathology alongside addressing the myofascial component to achieve effective relief [75].

Referred pain from MTrPs can mimic other conditions, leading to a cycle of chronic pain and muscle dysfunction. Referred pain is described in Table 3 [76].

**Table 3:** Critical symptoms of myofascial trigger points in different muscles that can cause chronic pelvic pain

Muscle	Critical symptoms
Piriformis muscle	Pain referred to the sacroiliac joint, buttock, and hip, which increases with standing and sitting. Entrapment of the sciatic nerve
Internal obturator muscle	Referred vulvar and urethral pain in women. Golf ball sensation in the rectum and/or vagina. Entrapment of the pudendal nerve
Ischiocavernosus and bulbocavernosus muscle	Perineal pain. Dyspareunia. Pain with orgasm. Clitoral pain
Levator ani muscle	Pain referred to the vagina. Golf ball sensation in the rectum or vagina. Post-defecation pain. Pain referred to the urethra and bladder

Management of MPS in pelvic pain involves a comprehensive approach. There are some perpetuating factors that can also play a significant role in the widespread distribution of referred pain through central sensitization mechanisms [77]. Mechanical issues, such as lower limb length discrepancy, postural issues, or gait disturbances, can cause continuous or prolonged myofascial stress, and without addressing these perpetuating factors, treatment will not be effective [76]. 

The approach may include several manual therapies such as the release of scar adhesions, myofascial release, desensitization of trigger points, deep transverse massage, mobilization techniques, and passive nerve stretching, as well as stretching of muscles with increased tone [41]. Thiele's massage can also be performed in patients with increased muscle tone. However, no RCT have been performed so far [78]. The 2022 EAU guidelines recommend offering biofeedback as an adjuvant therapy to muscle exercises for patients with anal pain due to an overactive pelvic floor, to relax the muscles [3]. Vaginal dilators are used to restore vaginal length, caliber, and myofascial extensibility [79]. Other options include TENS, ultrasound, and tecartherapy, though studies on these methods are limited [80-82]. 

Moldwin and Fariello suggest starting with conservative therapy for three months and recommended that BTA should be given no sooner than every three months [83]. If substantial improvement is not observed with conservative therapy, they recommend anesthetic infiltration as the next step, as both predictive and therapeutic measures [83]. Administration routes may include transvaginal or transperineal, depending on specific requirements and patient conditions. Moldwin and Fariello suggest using a minimal amount of anesthetic, typically no more than 0.25-0.5 ml per trigger point, and avoiding steroid injections in MTrPs due to the potential for muscle mass loss with repeated use [83].

Off-label use of BTA has shown mixed results in the treatment of MPS [34]. A recent retrospective cohort analysis demonstrated that administering injections followed immediately by rehabilitation significantly improved pain, likely due to enhanced tolerance for myofascial release directly after the injections [84].

Neuromodulation is still establishing its role in pelvic pain management. While there is growing evidence supporting its use, more detailed, high-quality research is needed [3]. Sacral neuromodulation has FDA approval for some urinary and fecal conditions. However, it is not FDA-approved for pain management [85]. A recent systematic review included 26 studies, and within this group, 13 studies reported significant pain improvement [85]. Thus, research is necessary to fully establish its efficacy and standardize its clinical use.

It was demonstrated by a systematic review that percutaneous tibial nerve stimulation is effective in reducing pain in patients with CPPPS [86]. Percutaneous tibial nerve stimulation is a less invasive technique, with good results in a short time with fewer side effects. However, we must consider that it has not been tested in the long term and results are lower if compared with sacral neuromodulation [87].

Pudendal neuralgia: neurological domain

The pudendal nerve is responsible for the development of CPP in 4% of the patients [88]. Pudendal neuralgia can be secondary to herpetic neuropathy, stretch neuropathy, and post-radiotherapy neuropathy, but pudendal nerve entrapment is by far the most common etiology [89]. Regarding entrapment, it can occur in the infrapiriform canal, between the sacrotuberous and sacrospinous ligaments (the most common cause), in Alcock's canal, and within the aponeurosis of the internal obturator muscle and may also affect the distal terminal branches [90].

Clinical characteristics include pelvic pain with sitting which increases throughout the day and decreases with standing or lying down, sexual dysfunction, and difficulty with urination and/or defecation [91]. This nerve can also be examined for irritability using the Tinel sign. This test is performed by lightly palpating the nerve within Alcock's canal. If light palpation causes sharp, shooting, stabbing pain or burning sensations, the Tinel sign is considered positive at this location [27].

In 2006, a multidisciplinary team held in Nantes, France, identified essential criteria for the diagnosis [92]. Firstly, pain should be limited to the innervation territory of the pudendal nerve. Secondly, pain is predominantly experienced while sitting, due to nerve compression etiology. In long-standing pudendal neuralgia, pain may become continuous, but it is still worsened by the sitting position. Thirdly, the pain rarely awakens the patient at night. Fourthly, on clinical examination, no objective sensory impairment can be found even in the presence of paresthesia. This can be explained in part by the sensory innervation of the perineum, in which there is an overlapping of different nerves. If a sensory deficit is found, a lesion at the level of the sacral roots or sacral plexus should be sought. Lastly, pain should be relieved by the anesthetic infiltration of the pudendal nerve [92].

There are also complementary diagnostic criteria: the sensation of a rectal foreign body and the worsening of pain during defecation, both of which should prompt the physician to exclude the differential diagnosis of chronic proctalgia. Exclusion criteria for pudendal neuralgia are pain in a territory unrelated to the pudendal nerve, symptomatic pruritus instead of paresthesia, and exclusively paroxysmal pain [92].

Imaging exams are not necessary for the diagnosis of pudendal neuralgia, but a pelvic magnetic resonance imaging or other tests are recommended to exclude any differential diagnosis.

As a treatment, it is recommended to avoid activities that cause hyperpressure, such as horseback riding or cycling, and use doughnut-shaped seat cushion [93]. As medications, the first-line treatments include tricyclic antidepressants, serotonin and norepinephrine reuptake inhibitors, or antiepileptics [93]. The medication of choice is antidepressants, specifically amitriptyline, although its use is not supported by high-quality studies [94]. Dosage can be increased based on the effectiveness and tolerability of the medication. Depending on the response, different medications can be used simultaneously to avoid very high doses that may cause side effects in the patient. Most patients have slight to moderate improvement in pain with noninvasive treatment modalities [88].

When neuropathy is associated with MPS, particularly at the level of the levator ani muscle and the internal obturator muscle, rehabilitation is recommended as described above.

Pudendal nerve infiltration is another option. It has been shown to have good short-term effects but lacks efficiency in the long term [95]. Also, recommendations from the European Journal of Pain in 2021 conclude that pulsed radiofrequency is effective in the context of pudendal neuralgia, although it is not considered a first-line treatment [93]. About 79% of patients were considered as "fairly or much better" three months after the technique, and in 89% of patients, this improvement was maintained at follow-up with a median of four years [88]. 

Decompression surgery is considered a second-line treatment but yields good results [51]. Neuromodulation, although supported by limited literature, is used in cases where surgical decompression is not possible.

## Conclusions

CPPPS represent a significant challenge in women's health due to their multifactorial nature and the absence of a definitive pathophysiological mechanism. Many patients still face prolonged suffering due to delayed diagnosis and suboptimal treatment. The existing studies on various treatments are not robust, highlighting the necessity for further, more comprehensive research. Thus, continued research is essential to better understand the underlying mechanisms of these conditions and to develop more effective and targeted therapies. Multidisciplinary collaboration remains key to improving outcomes for women suffering from these debilitating conditions.
